# Improving ChIP-seq peak-calling for functional co-regulator binding by integrating multiple sources of biological information

**DOI:** 10.1186/1471-2164-13-S1-S1

**Published:** 2012-01-17

**Authors:** Hatice Ulku Osmanbeyoglu, Ryan J Hartmaier, Steffi Oesterreich, Xinghua Lu

**Affiliations:** 1 Department of Biomedical Informatics, University of Pittsburgh School of Medicine, Pittsburgh, PA, USA; 2 Department of Pharmacology and Chemical Biology, University of Pittsburgh School of Medicine, Pittsburgh, PA, USA

## Abstract

**Background:**

Chromatin immunoprecipitation coupled with massively parallel sequencing (ChIP-seq) is increasingly being applied to study genome-wide binding sites of transcription factors. There is an increasing interest in understanding the mechanism of action of co-regulator proteins, which do not bind DNA directly, but exert their effects by binding to transcription factors such as the estrogen receptor (ER). However, due to the nature of detecting indirect protein-DNA interaction, ChIP-seq signals from co-regulators can be relatively weak and thus biologically meaningful interactions remain difficult to identify.

**Results:**

In this study, we investigated and compared different statistical and machine learning approaches including unsupervised, supervised, and semi-supervised classification (self-training) approaches to integrate multiple types of genomic and transcriptomic information derived from our experiments and public database to overcome difficulty of identifying functional DNA binding sites of the co-regulator SRC-1 in the context of estrogen response. Our results indicate that supervised learning with naïve Bayes algorithm significantly enhances peak calling of weak ChIP-seq signals and outperforms other machine learning algorithms. Our integrative approach revealed many potential ERα/SRC-1 DNA binding sites that would otherwise be missed by conventional peak calling algorithms with default settings.

**Conclusions:**

Our results indicate that a supervised classification approach enables one to utilize limited amounts of prior knowledge together with multiple types of biological data to enhance the sensitivity and specificity of the identification of DNA binding sites from co-regulator proteins.

## Background

Transcription factors (TFs) serve as the final molecules in signal transduction pathways that coordinate expression of target genes. When activated in response to upstream signals, often encoded as chemical ligands and protein modification, TFs bind to their cis-regulatory sites to exert their regulatory effects on their target genes. During the process, TFs often interact with other proteins, which further modulate the function and efficacy of TFs to achieve fine-tuned regulation of gene expression; studying such interactions and regulations is an increasingly important component of studying gene expression systems. Nuclear receptors (NRs), such as estrogen receptor α (ERα), are transcription factors that migrate to the nucleus (often as a result of binding ligand) to regulate downstream target genes. NRs play important biological roles in normal physiology and disease. In particular ERα plays an important role in both breast cancer and osteoporosis. Upon ligand binding, ERα and other NRs are bound by proteins called co-regulators that recruit transcriptional machinery and chromatin modifying enzymes. Co-regulators are therefore critical in NR activity. Understanding the composition of functional NR/co-regulator complexes in specific signaling contexts could provide a basis for the development of novel NR- and co-regulator-targeted therapeutics. The problem addressed in this paper arose from a study of the interaction between the major ERα co-activator SRC-1 (a member of the p160 SRC family), also known as NCOA1, with ERα and the impact of such interactions gene expression [1-4].

Recently, chromatin immunoprecipitation coupled with high-throughput next-generation sequencing (ChIP-seq) has become the main technology for global characterization of the transcriptional impact of NRs and their co-regulators [5-7]. ChIP-seq involves the short-read (~30 bp) sequencing of the ChIP-enriched DNA fragments. These short sequence reads (tags) are then aligned to a reference genome. Then the actual binding loci from the positional tag distributions (i.e. sequenced DNA fragments mapped onto a reference genome sequence) are determined using 'peak calling' algorithms. Numerous peak calling algorithms have recently been developed for identifying ChIP-enriched genomic regions from ChIP-seq experiments [8-10] but there is a wide range of discordance among the peak calls from different algorithms [11]. Therefore, there is a need for the methods that can integrate additional information besides ChIP-seq tags to identify functional TF binding sites. Furthermore, studying the interactions between TFs and their co-regulators through ChIP-seq technology poses an additional challenge since co-regulators do not directly bind DNA. Co-regulator ChIP-seq measures the secondary protein-DNA binding through primary TFs and leads to relatively weak sequencing signals--i.e. relatively small number of sequence tags above noise. As such, it remains a challenge for contemporary peak calling methods to detect weak secondary protein-DNA-binding signals and simultaneously maintain a high specificity.

Often, a well-designed experiment studying interaction between a TF and its co-regulator generates critical information in addition to the ChIP-seq data for the co-regulator binding. For example, ChIP-seq data reflecting the binding of the primary TF of interest to its cis-regulatory sites are often collected; the genomic sequence surrounding Chip-seq peaks are usually available, which can be used to reflect the intrinsic sequence characteristics of regulatory sites; transcriptomic data that reflect functional outcomes of the interaction of the TF and its co-regulators can also be monitored. In this study, we investigated and compared different statistical and machine learning approaches to integrate multiple types of information to overcome the difficulty of identifying functional ERα/SRC-1 interaction in presence of weak ChIP-seq signal.

## Results and discussion

The biological study underlying this paper aims to investigate the impact of ERα/SRC-1 interaction on estrogen induced gene expression in a bone cell line transfected with ERα (U2OS-ERα), which may shed light on the effect of estrogen-related bone development, bone loss, and potentially bone metastasis. We have generated ChIP-seq data using anti-ERα and anti-SRC-1 antibodies in presence and absence of estradiol (E2). To further investigate the impact of interactions between this NR//co-regulator pair, we collected expression array data from the same cell lines with a combination of E2 treatment and SRC-1 knock down. In general, the results of an SRC-1 ChIP-seq experiment would reflect secondary, indirect binding of SRC-1 to DNA through multiple NRs. However, in this study our experimental design aims to investigate specifically estrogen-induced interactions between ERα, SRC-1, and DNA. The detailed results of the experiments are being prepared for a separate publication (Hartmaier et al., manuscript in preparation). In the current paper, we address the fundamental issue of identifying reliable and functional ERα/SRC-1 DNA binding sites.

### Identifying SRC-1 binding sites based on anti-SRC-1 ChIP-seq

We first set out to investigate the efficacy of studying ERα/SRC-1 DNA binding sites only based on the results of ChIP-seq experiments performed using an anti-SRC-1 antibody. Potential SRC-1 binding peaks were identified using three different algorithms: MACS 1.4.1 [9], BayesPeak[10], and T-PIC [8]. Table 1 shows the number of peaks identified by the above algorithms with different cut-off thresholds and the corresponding number of genes to which the peaks are mapped.

**Table 1:** The number of peaks called by different algorithms and at thresholds, and corresponding number of mapped genes

Method	Total number of peaks	Number of genes mapped
MACS, p = 1E-8	1,966	996
MACS, p = 1E-5	4,678	2,054
MACS, p = 1E-3	23,306	6,341
T-PIC, p = 1E-3	4,453	1,676
T-PIC, p = 1E-2	6,598	2,318
BayesPeak (PP = 0.90)	15,622	4,495
BayesPeak (PP = 0.70)	21,373	5,507
BayesPeak (PP = 0.5)	27,990	6,533
Union*	38,324	8,057
Intersection*	4,811	2,029

**Figure 1:**
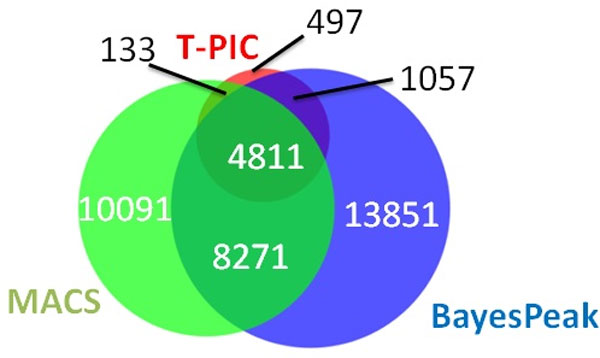
**Peak calling by different algorithms.** A Venn diagram shows the overlaps among the peaks called by MACS (*P *value cutoff of 10^-3^), T-PIC (*P *value cutoff of 10^-2^) and BayesPeak (*PP *cutoff of 0.5). The number of peaks are shown. The numbers of the union and intersection of the peaks and the mapped genes by the algorithms are shown in Table 1.

* Union and intersection of the peaks by the three methods, as shown in Figure 1.

The results of the table raise the following issues during interpretation: First, as expected, applying different cut-off thresholds to the results by a given algorithm leads to a different number of peaks being identified: there is inevitably a trade-off between the number of peaks recovered and the quality of the peaks. Second, different algorithms assess the quality of the peaks based on different assumptions and methodologies: there is no consensus on the "goodness" of quality scores of these algorithms. We further noted that different versions of a same algorithm return different quality scores. Finally, different algorithms return disjointed sets of peaks, as shown in Figure 1, indicating that distinct assumptions and approaches enable an algorithm to discover some potential peaks that evade detection by other algorithms. These issues force decisions that potentially impact the conclusions of the study such as: which algorithm performs better, what cut-off threshold for a given algorithm to pick, and how to consolidate the results from different algorithms so that one can maximize the number of high quality peaks. Making these choices remains challenging due to the lack of consensus in the field [11,12].

In order to compare our results with a recently published study by Lanz et al. [13], which analyzed DNA recruitment of the co-regulators SRC-3, we studied the estrogen-induced SRC-1 peaks identified from our data using MACS algorithm with a cut-off threshold of *P *value at 10^-10^, a threshold based on their study. Our analysis yielded a total of 1,286 peaks, which were further mapped to 684 genes. The number of peaks identified by us with the above condition is far fewer compared to their study. The discrepancy is likely to be, at least in part, due to amplification of SRC-3 in MCF-7 cells used in their study and possible differences in antibody affinities. However, these results also raised the hypothesis that ChIP-seq signals of secondary binding at physiologic levels are usually weaker. This suggests that conventional cutoff thresholds for peak calling algorithms may be too stringent, neglecting weak peaks (peaks with relatively small number of tags) potentially resulting from real ERα/SRC-1/DNA interactions. Therefore, additional information besides SRC-1 ChIP-seq tags should be capitalized to enhance identification of functional binding sites.

### Integrating multiple sources of biological information for identifying SRC-1 binding sites

To corroborate the results of SRC-1 ChIP-seq, we also studied the ERα ChIP-seq data (reflecting the expected dominant SRC-1-interacting TF) and investigated peaks overlapping between the ERα and the SRC-1 ChIP-seq results. By varying the cut-off threshold of MACS, we identified different numbers of overlapping peaks between ERα and SRC-1, with the number of overlapping peaks increasing as the cut-off threshold relaxes (data not shown). The results again indicated that the conventional cut-off thresholds are failing to identify putative ERα/SRC-1 DNA binding sites (false negatives). On the other hand, simply relaxing the cut-off threshold is likely leading to increased false positive peak calls. Thus a principled method is needed to further identify functional ERα/SRC-1 DNA binding sites.

To elucidate *functional *ERα/SRC-1 DNA binding events, i.e., the binding events that influence gene expression, we generated and analyzed expression array data from cells that were treated with vehicle and E2 in the presence and absence of SRC-1 siRNA (Hartmaier et al., manuscript in preparation). The microarray data enabled us to identify 634 genes whose expression response to estrogen treatment required SRC-1, which are hereto referred to as SRC-1-sensitive genes. When we compared the list of SRC-1-sensitive genes and the list of genes with SRC-1 binding sites derived by MACS at *P *value = 10^-10^, we noted that only 44 genes overlapped among the lists. While the discrepancy between the number of SRC-1-sensitive genes and genes with SRC-1 peaks could be explained by other biological factors, such as that many SRC-1 peaks were not functional or secondary expression effects, it also supported our general hypothesis that ERα/SRC-1 interaction ChIP-seq signal is relatively weak and potentially true functional ERα/SRC-1 DNA binding sites were missed by the stringent setting of the peak calling algorithm.

While it may be tempting to directly combine the information from SRC-1 ChIP-seq, ERα ChIP-seq and microarray data by identifying the intersections of overlapping genes and peaks, such an approach is overly simplistic and ignores other potentially informative data, e.g., the genome-sequence characteristics of ERα/SRC-1 interaction sites and the prior information of known ERα/SRC-1 interactions. These considerations motivated us to investigate and compare different principled machine learning approaches in order to improve the sensitivity and specificity of detecting ERα/SRC-1 DNA binding sites by integrating multiple types of information.

### An integrative approach to detect ERα/SRC-1 DNA binding sites

The overall framework and rationale of our information integration approaches are as follows. We formulated the task of identifying functional ERα/SRC-1 DNA binding sites as a classification task, in which we performed a binary classification to label a potential SRC-1 binding site derived from ChIP-seq analysis as either functional or nonfunctional. We investigated both supervised learning, which allows us to take advantage of existing knowledge of ERα/SRC-1 interactions, and unsupervised learning, which allowed us to take an unbiased approach.

The classification formulation allowed us to pool more candidate peaks identified by different peak calling algorithms at relaxed cutoff thresholds so that we did not have to rely on a single "best" algorithms and "optimal" parameterization but resorted to our classification to identify functional ERα/SRC-1 DNA binding. In this study, we collected the union of the peaks returned by all three algorithms at the cutoff threshold as follows, MACS: *P *value cutoff 10^-3^, BayesPeak: Posterior Probability (PP) ≥ 0.5 and T-PIC: *P *value cutoff 10^-2^. This led to a pool of 38,324 candidate peaks.

Another important advantage of the classification approach is that it allows us to integrate multiple types of biological information collected from our experiments and public databases by representing them as features for a classifier. In this way, multiple types of information contribute to the classification of potential peaks and their impact can be determined by learning algorithms. For each candidate SRC-1 peak, we constructed the following features: a vector of binary features representing presence/absence of nucleotide trigrams (triplet of nucleotides), which reflects the intrinsic characteristics of genome sequence surrounding the summit of a peak region; an average of predicted nucleosome-occupancy scores, which represents the chromatin structure characteristics around the peak summit; a binary feature reflecting if a primary binding peak, i.e., the ERα ChIP-seq peak, overlaps with the SRC-1 peak; and a binary feature representing the functional outcome of ERα/SRC-1 interaction, i.e., whether the peak is mapped to an SRC-1 sensitive gene. Detailed descriptions of features are presented in Methods section.

We evaluated the results of predictions from classification algorithms by determining if conserved ERα binding motif can be found in the classified peaks, as an indication that a peak is the result of ERα/SRC-1 DNA binding. Searching for instances of conserved TF binding motifs at the predicted binding loci is considered the most prominent verification method for validating peaks [14].

### Unsupervised classification

First, we explored if the candidate peaks could be divided into two distinct groups by unsupervised learning in an unbiased manner. We applied a K-means clustering procedure to the data and the results are listed in Table 2. We inspected the genome sequences of the peaks to assess if a conserved motif for estrogen response element (ERE) was detected by a motif classification algorithm referred to as CLOVER[15]. In cluster 46% of 26,211 peaks contained the ERE motif, and, in cluster 27% of 12,113 peaks contained the ERE motif. We believe this is not a good separation of the peaks in that, even though cluster 1 has more ERE-containing peaks, it is a bigger cluster and only 46% of peaks contain EREs. Thus the results would likely lead to a high false positive rate with respect to SRC-1 binding.

**Table 2:** Comparison of the performances by different machine learning algorithms

	Number of peaks	Number of peaks with ERE motif	Ratio of peaks with ERE motif match
MACS p = 1E-10	1,286	941	0.73
MACS p = 1E-8	1,966	1,416	0.72
MACS p = 1E-5	4,678	3,077	0.66

k-means (city block)			

Cluster 1	26,211	11,943	0.46
Cluster 2	12,113	3,245	0.27

supervised-NB(th = 0.8,1:2)			

Positively labeled	11,835	8,196	0.69
Negatively labeled	26,489	6,992	0.26

supervised-SVM(kernel = polynomal,1:2)			

Positively labeled	14,915	8,425	0.56
Negatively labeled	23,409	6,763	0.29

supervised-RF(th = 0.7,1:2)			

Positively labeled	10,428	6,514	0.62
Negatively labeled	27,896	8,674	0.31

semi-supervised-NB(th = 0.8,1:2, I = 75)			

Positively labeled	12,597	8,458	0.67
Negatively labeled	25,727	6,730	0.26

### Supervised classification

Supervised learning requires labeled data as training cases. Obtaining a training set through large-scale experimental validation of ERα/SRC-1 DNA binding is costly and difficult to perform. Therefore, we investigated whether a relatively small amount of labeled data in the supervised learning task would result in better separation compared to unsupervised clustering with our feature set. We experimentally validated 18 SRC-1 peaks as functional peaks by quantitative PCR (qPCR) experiment (data not shown). We used these peaks as positive training cases, together with a set of randomly drawn control (anti-IgG) ChIP-seq peaks as negative training cases, to train supervised classifiers. We investigated the performance of three state-of-the-art classifiers: Naive Bayes (NB), Support Vector Machines (SVM) and Random Forest (RF). For NB and Random Forest classifiers, we set the classification thresholds at 0.8 and 0.7 respectively. Since the ratio of the positive and negative training cases may have impact on classification algorithms, e.g. NB and SVM, we explored using different ratios for training, between 1:1, 1:2, and 1:3, and classifiers were built from these training sets. Our test set consisted of all 38,324 candidate peaks. Table 2 lists the total number of peaks in each class, the number ERE-containing peaks in each class and the ratio reflecting ERE enrichment. Results for classifiers with 1:1, 1:2, and 1:3 training ratio (positive over negative) were very similar to each other (data not shown). Therefore, just results for the 1:2 ratio were shown.

We noted that the supervised classification approaches have significantly increased the number of positive peaks when compared to those derived by peak calling algorithms based on recommended cutoff thresholds. For example, NB returned 11,835 positive peaks in comparison to 1,966 and 4,678 peaks returned by MACS with cutoff *P *value set at 1E-8 and 1E-5, which reflected a 6-fold and 2.5-fold increase, respectively. Through further evaluation enrichment of ERE in the genome sequences surrounding the peaks, we found that a similar percentage of peaks contained ERE element: 69% for NB, and 72% and 66% MACS at 1E-8 and 1E-5 respectively. Thus, the results indicate that the qualities of the positive peaks returned by NB were as good as those returned by the stringent peak calling in terms of ERE enrichment.

We further inspected if the classification approaches retrieved additional *functional *peaks, i.e., the peaks that were mapped to SRC-1-sensitive genes derived from microarray experiment. Table 3 shows the results of the SRC-1 peaks returned by different peak calling approaches that were mapped to SRC-1-sensitive genes. We noted that, by setting MACS cutoff *P *values at 1E-10, 1E-8, and 1E-5, a total of 44, 57, and 123 peaks were mapped to SRC-1-sensitive genes. On the other hand, NB has identified 238 peaks that overlap with SRC-1-sensitive genes. We also note that BayesPeak exclusively identified some of the newly "discovered" functional peaks. Similarly MACS also exclusively discovered some new peaks. These results indicate that, based on different assumptions and criteria, different peak calling algorithms are capable of identifying potential peaks to complement other peak calling algorithms. Thus it is more sensible to consider candidate peaks from more than one peak-calling algorithm as long as an objective approach can be further applied to consolidate the results.

**Table 3:** Comparison of different methods for identifying functional peaks

Method	Total number of peaks	Number of genes mapped	Intersection with SRC1-dependent genes
MACS p = 1E-10	1,286	684	44
MACS p = 1E-8	1,966	996	57
MACS p = 1E-5	4,678	2,054	123
supervised-NB(th = 0.8,1:2)	11,835	3,875	238

We also performed a 9-fold cross-validation experiment to assess if the algorithm can correctly identify the experimentally validated ERα/SRC-1 training cases from the candidate peak pool. Results were listed in Table 4. NB classifier showed 89% precision, 100% recall and 96% accuracy, see Methods section for the descriptions of the metrics. This result increased our confidence that positive calls from our algorithm are likely to reflect real ERα/SRC-1 DNA binding.The results in the table indicate that the NB classifier performed better than the SVM and RF classifiers, judging from relative enrichment of ERE containing binding sites in the predicted positive peaks. Among the three classifiers tested in this study, the RF classifier performed worst. We noted that the number of features that were used by RF during the learning was much smaller than the number of features utilized by other classifiers, which may partially explain the inferior performance of this algorithm in this experiment. The SVM method also performed worse on this task than probabilistic NB. We conjecture that the reason might be that SVM is complex algorithm with many parameters to adjust and therefore finding optimal parameters for decision boundary might be challenging for this task. We therefore concentrated on the NB classifier because it could be readily used in both supervised and semi-supervised learning environment.

**Table 4:** Performance of different classifiers under 9-fold cross-validation setting

Classifier	Precision	Recall	Accuracy
NB(th = 0.8,1:2)	0.89	1	0.96

SVM(kernel = polynomial,1:2)	0.89	0.96	0.94

RF(th = 0.7,1:2)	0.72	1	0.91

### Semi-supervised classification

Our number of training cases is relatively sparse compared to a conventional machine learning setting. Semi-supervised approaches have been applied to conquer limitations of supervised and unsupervised methods when labeled data is scarce and obtaining large amounts of labeled data is expensive and time consuming [16,17]. This is done by incrementally assigning instances, which are called with high confidence by a classifier, from unlabelled data into training cases in order to increase the number of training cases and thus enhance the generalizability of classification. Therefore, we investigated semi-supervised classification to see whether we could further increase the performance in identifying ERα/SRC-1 DNA binding.

**Figure 2:**
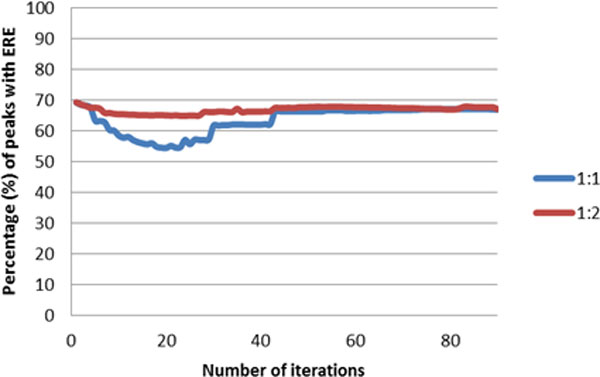
**Self-training.** Percentage of predicted positive peaks with ERE motifs (over iterations for different TP:TN ratios for training set as indicated in the legends).

In this study, we applied a self-training algorithm [17] using NB as the base classifier because of its probabilistic outputs. We iteratively assigned the most confident positive instances called by our classifier into training cases and found that performance of the self-training became stable after 75 iterations and stopped further training. Figure 2 shows the trends of percentage of ERE-containing peaks in the positive calls in semi-supervised learning. It is interesting to note that initially as a few pseudo-positive cases were imputed into the training cases the precision of the called positive peaks decreased but later became stable after 75 iterations. A similar total number of peaks and the percentage of ERE-containing peaks were identified by our semi-supervised learning algorithm when compared to other supervised learning experiments, see Table 2. Thus the results do not show obvious advantage of semi-supervised learning over supervised learning algorithms in our experiment.

### Identification of informative features

**Figure 3:**
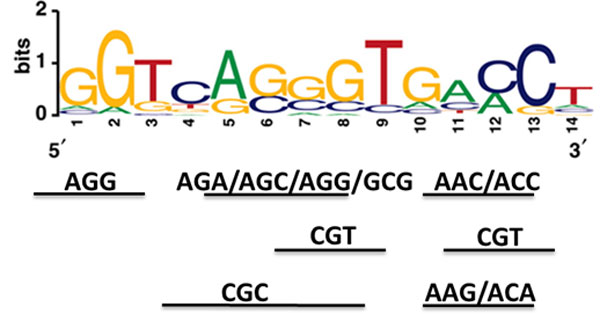
**Overlapping top trigrams with ERE motif.** This figure shows potential matching locations of the top-ranking nucleotide trigrams identified by feature selection algorithms.

Biologically, it is of interest to identify the features that significantly contribute to the classification in that it will reveal the relationships between input features and outcome. We rank key features by ROC class reparability criteria using MATLAB (Bioinformatics Toolbox) [18], using a training dataset containing the 18 true positive peaks and 36 random non-binding sites from IgG peak calls. Following were the 15 top ranked features: *"AAC", peaks-mapped-to-SRC-1-dependent-genes, "GCG", "CGT", overlapping-with-ERα-peak, "AAG", "ACA", "ACC", "CGC", "AGA", "AGC", "AGG", "CGA", "ACT", "ATC"*, see Methods section for detailed descriptions of the features. Among the top-ranking features, we noted that the features reflecting the function outcome (*peaks-mapped-to-SRC-1-dependent-genes*) and the interaction between ER and SRC-1 (*overlapping-with-ERα-peak*) were ranked high, indicating the learning algorithm correctly recognized their importance in classification. It is interesting to note that many nucleotide trigrams, which reflect the characteristics of sequences of peaks, were among the high-ranking features. We aligned the top-ranking trigrams to the ERE motif, as shown in Figure 3. Indeed, the trigrams correspond well with the important components of the ERE motif. These results indicate that the classification learning algorithm, like the motif searching algorithms (i.e. [19-22]), is able to identify highly conserved "words" that constitute one of the important motifs of the training sequences. We noted that the feature reflecting nucleosome occupancy at peak regions was ranked as 48^th^. This may indicate either that nucleosome occupancy is a dynamic process and our static feature does not reflect the true occupancy status during the experiments or that the ERa/SRC-1 DNA binding is not heavily dependent on nucleosome occupancy.

### Biological insights from improved peak calling

We further examined the impact of the improved SRC-1 peak calling on biological insights drawn from the dataset. We conducted Gene Ontology Analysis using the Database for Annotation, Visualization, and Integrated Discovery (DAVID) [23] on genes with an SRC-1 peak within 50 kb of the TSS as determined by MACS (P < 10^-5^) or by our method. We observed a dramatic difference in the identification of genes enriched in specific biological processes. Specifically, our method yielded in the calling of peaks in gene sets which were highly enriched for genes involved in blood vessel development (enrichment: 4.61, Benjamani: 7.9 × 10^-4^) and actin filament-based processes (enrichment: 3.18, Benjamani: 1.3 × 10^-3^). Indeed blood vessel development has previous been implicated in bone generation [24,25]. Further, within the genes enriched in these biological processes, we identified a number of genes with known functions in bone development. Since SRC-1 has already been implicated in E2 mediate bone maintenance, this observation provides evidence for the mechanism underlying this phenotype. In contrast, genes with SRC-1 peaks determined by MACS were not significantly associated with any biological processes.

## Conclusions

We believe that the ability to improve ChIP-seq peak calling by utilizing available sources of biological information for indirect co-regulator binding in the presence of weak ChIP-seq signal is an important research area. Due to the intrinsic variability in the affinity of interactions between a TF and its co-regulators, it is inevitable that the ChIP-seq signal of these types of studies would span a broad spectrum and that the weak signal scenario, as in this study, would be likely to occur often. The need for methods to address this problem is acute considering the increasing number of studies using ChIP-seq to study NR and their co-regulators due to their importance in normal development and in many diseases such as breast cancers. Our work strives to explore whether the peak calling can be improved through the integration of available diverse biological sources via machine learning approaches. Our results demonstrate that it is informative to generate, collect, and integrate the following information: ChIP-seq data reflecting location of the primary interaction of the TF of interest to its cis-regulatory sites, gene expression data reflecting functional outcomes of interaction of the TF and its co-regulators, and finally genomic sequence data of the identified regions. Other types of data which is highly likely to be useful include histone modification marks, recruitment of RNA polymerase II, and relative location of the insulator protein CTCF [26].

In summary, our results indicate that a supervised classification approach enables one to utilize even limited amounts of existing knowledge together with multiple types of biological data to enhance the sensitivity and specificity of identifying DNA binding sites for co-regulators proteins. Our feature selection experiments indicate that experimental inputs complementary to ChIP-seq are critical in identifying biological significant signals from ChIP studies with weak signals due to indirect DNA binding.

## Methods

### ChIP-seq data

U2OS cells stably expressing Flag-tagged ERα (obtained from Dale Leitman) were used for ChIP as previously published [27]. SRC-1 and ERα ChIP DNA from ethanol (vehicle) and estradiol (E2)-treated U2OS cells were amplified for Illumina sequencing. IgG ChIP DNA was also amplified for Illumina sequencing. The ChIP-seq datasets used in this study had the following number of uniquely mapped sequence tags, ChIP_ER_E2: 10,380,852, ChIP_SRC-1_E2:6,995,566 tags, ChIP_IgG: 8,641,543 tags. SRC-1 peaks were called using MACS 1.4.1 [9], BayesPeak[10], and T-PIC [8] with IgG as negative control.

### Evaluation procedure

The selected peaks were evaluated in terms of their overlap with high-scoring sequence motifs. The motif analysis was performed using the program CLOVER [15], with *P *value cutoff 0.005 which compares sets of DNA sequences to a library of transcription factor-binding motifs and identifies whether any of the motifs are statistically overrepresented or underrepresented in the sets. We measured enrichment of selected motifs in sets of ± 300 bp from SRC-1 ChIP-seq peak summit.

### Computational framework

We investigated the following computational approaches to identify potential binding sites, including unsupervised classification, supervised classification and semi-supervised classification. The task was formulated as a binary classification problem for supervised and semi-supervised framework, where each ChIP-seq peak was either 'functional' or 'non-functional'. Each ChIP-seq peak was represented with a vector of binary features, where each feature was derived from one biological information source.

### Features

We devised a total of 67 features, which can be grouped as follows. 1) Genomic information: trigrams (triplet of nucleotides) to represent intrinsic characteristics of genome sequence surround the peak summit to create a feature vector; averaged nucleosome occupancy prediction results as another feature. 2) Primary TF binding events: the called ER ChIP-seq data peak that overlap with SRC-1/ERαChIP-seq. 3) Functional outcome of TF activation: whether the peak is mapped to SRC-1 sensitive gene.

### N-gram presence (64 features)

Previously, n-gram distribution of sequences have been utilized for TF binding site prediction [28]. An N-grams consists of a sequence of *n *letters, where letters are possible nucleotide (A,T,G,C) bases of DNA sequences in ChIP-seq peaks. As such, a trigram has 64 possible combinations of three nucleotides, and we constructed a vector of length of 64 elements used the Galaxy Toolkit [29], each representing the presence or absence of a give trigram in the 600 bp surrounding the summit of the peak of interest.

### Nucleosome occupancy (1 feature)

Nucleosomes are fundamental repeating unit of eukaryotic chromatin. Nucleosomes consist of 147 bp of DNA sequence wrapped around a histone core complex,and they are separated from each other by linker DNA of up to 50 bp. Recently, Tillo et al. [30] proposed that nucleosome occupancy of DNA sequence around functional human transcription factor binding sites (TFBSs) is remarkably higher. To represent the nucleosome occupancy status of the Chip-seq peaks, we use the scores from Kaplan et al.'s [31] genome wide nucleosome predictions. For each base location of human genome, Kaplan et al. provided the "average occupancy" score, which is the predicted probability for each position in the genome to be covered by any nucleosome. For each peak, we took the mean value of average occupancy score around ±50 bp (an approximate length of a nucleosome) region of the peak summit. For each candidate peak, its nucleosome occupancy feature is represented as a binary variable, with value set equal 1 if the mean value greater than 0.75 and 0, otherwise.

### Primary TF binding events (1 feature)

For each candidate SRC-1 peak, we associate a binary variable to indicate if the peak overlaps with any ERα ChIP-seq peak. We defined that an ERα and an SRC-1 peak overlap if they share at least one base pair.

### Functional outcome of TF activation (1 feature)

We collected the gene expression data from cells that were treated with vehicle and E2 in presence and absence of anti-SRC-1 siRNA have been employed for our analysis. Differentially expressed genes between these samples were found using limma (Linear Models for Microarray Analysis) package - an implementation of the empirical Bayes linear modelling approach [32]. We identified a list of genes that were differentially expressed between the control vs anti-SRC-1 siRNA groups and labelled them as SRC-1 sensitive genes. *ChIPpeakAnno*[33] was used to map each ChIP-seq peak to a gene if possible using default setting of the program. For each candidate SRC-1 peak, we associate a binary variable to indicate if the peak is mapped to one of SRC-1-sensitive genes.

### Machine learning approaches

For unsupervised learning, k-means clustering, training and classification procedures for supervised and semi-supervised framework are implemented using the MATLAB^®^ (Natick, MA). We rank key features by ROC class reparability criteria using also MATLAB^®^. The microarray data analysis was done with the use of the R packages from the Bioconductor project http://www.bioconductor.org. We used DAVID [23] for GO analysis.

### Unsupervised clustering

We used k-means clustering (k = 2) with city block distance metric to see cluster candidate peaks into two groups.

### Supervised classification

To build this type of classifiers, labelled data of both true-positive peaks and false-positive peaks were required. We experimentally validated 18 SRC-1 peak by quantitative PCR (qPCR) experiments (data not shown), which were used as positive training cases, together with a set of randomly drawn control (anti-IgG) ChIP-seq peaks as negative training cases, to train supervised classifiers. We investigated the performance of three state-of-the-art classifiers: Naive Bayes (NB) [34] implemented by the MATLAB, Support Vector Machines (SVM)[35] and Random Forest (RF)[36]. Different ratios of positive to negative cases, (1:1, 1:2 and 1:3), were considered in this study for testing, and training.

NB classifier with *Bernoulli *distribution was used where each peak represented as binary-valued feature vectors. For SVM, we studied different types of kernels and chose the polynomial kernel in this study. For training RF classifiers, we grew 50 trees. For the number of variables randomly selected at each node, we used the default value that was equal to the square root of the feature dimension.

We measured performance of classifiers with 9-fold cross-validation process and report precision, recall and accuracy values. Precision and recall were used in order to evaluate model performance of classifier. Precision was measured as the fraction of correctly predicted TP binding sites (experimentally verified) among all binding sites predicted by the classifier to be TP binding site. Recall is the fraction of the TP binding sites that are also predicted to TP. Accuracy is calculated as the fraction of correct calls (TP + TN) overall total number of predictions.

### Semi-supervised classification

Self-training is one of the common algorithms used for semi-supervised learning [16]. In self-training [17], a classifier is built from labeled instances (*L*) and used to predict the labels for instances in unlabeled set (*U*). Then *m *instances in *U *that the current classifier has high classification confidence are labeled and moved to enlarge *L*. The whole process iterates until stopped. The stopping criterion in self-training is that, either there is no unlabeled instance left or the maximum number of iterations has been reached. Different ratios of positive to negative cases, (1:1, 1:2), were considered in this study for testing, and training. The detailed algorithm is shown below.

## Algorithm

Input: positively labeled data (*P*) ${\left\{\left(x_{i},y_{i}\right)\right\}}_{i=1}^{lp}$, negatively labeled data (*N*) ${\left\{\left(x_{j},y_{j}\right)\right\}}_{j=1}^{ln}$, and unlabeled data (*U*) ${\left\{x_{k}\right\}}_{k=lp+ln+1}^{lp+ln+u}$

1. Initially, let $L_{0}=\;{\left\{\left(x_{i\;},\;y_{i}\right)\right\}}_{i=1}^{lp}\cup \text{​}\;{\left\{\left(x_{j\;},\;y_{j}\right)\right\}}_{j=1}^{ln}$ and $U=\;{\left\{x_{k}\right\}}_{k=lp+ln+1}^{lp+ln+u}$ where *l_n _*= *l_p_*.

2. Set t, the iteration counter, to 0.

3. Repeat until the stopping criteria are not satisfied,

a. Build a classifier *C_t _*on *L_t_*.

b. Apply *C_t _*to the unlabeled instances in *U_t _*to predict a label for each instance in *U_t_*.

c. Generate $L_{t}^{s}$ by selecting unlabeled instances that *C_t _*has the highest classification confidence as positive label and select randomly equal number of negatively labeled instances from *N_t_*.

d. Delete the selected instances positively and negatively labeled from *U_t _*and *N_t _*respectively.

e. $L_{t+1}=L_{t}+L_{t}^{s}$.

f. Increase t by 1.

Return the final classifier and apply it to the *U*.

## Competing interests

The authors declare that they have no competing interests.

## Authors' contributions

SO and RH conceived, designed and carried out the biological experiments and ChIP-seq experiments. HUO and LX conceived and designed the computational analysis. HUO and LX drafted the manuscript and all authors participated discussion and editing of the manuscript.
